# Inverse Neural Network Approach for Optimizing Chemical Composition in Shielded Metal Arc Weld Metals

**DOI:** 10.3390/ma18112592

**Published:** 2025-06-01

**Authors:** Taehyun Yoon, Young IL Park, Jaewoong Kim, Jeong-Hwan Kim

**Affiliations:** 1 Department of Naval Architecture and Offshore Engineering, Dong-A University, Busan 49315, Republic of Korea; yoon.th2718@gmail.com (T.Y.); parkyi1973@dau.ac.kr (Y.I.P.); 2 Gwangju Ppuri Technology Support Center, Korea Institute of Industrial Technology, Gwangju 11109, Republic of Korea

**Keywords:** inverse neural network, shielded metal arc weld, genetic algorithm, chemical composition

## Abstract

This study presents a hybrid machine learning framework combining an artificial neural network and a genetic algorithm to optimize chemical compositions of shielded metal arc weld metals for achieving targeted mechanical properties. First, a neural network model was trained using a large experimental database provided by Dr. Glyn M. Evans, which includes the chemical compositions and mechanical properties of over 950 shielded metal arc weld metals. The neural network model, optimized via Bayesian optimization, demonstrated high predictive accuracy for properties such as yield strength, ultimate tensile strength, and Charpy impact transition temperatures. To enable inverse design, a genetic algorithm-based optimization was applied to the trained neural network model, iteratively exploring the composition space to find optimal elemental combinations that match predefined mechanical property targets. The proposed hybrid approach successfully identified multiple feasible compositions that closely match the desired mechanical behavior, demonstrating the potential of neural network-assisted inverse design in welding alloy development.

## 1. Introduction

The mechanical properties of welds are significantly influenced by their chemical composition, which has led to extensive research to understand this relationship. Mechanical tests have been conducted on various materials and welding methods, resulting in the development of a comprehensive database that links chemical composition to the mechanical properties of welds [1,2,3,4,5]. In May 2015, Dr. Glyn M. Evans published an extensive database on ResearchGate, encompassing over 950 shielded metal arc (SMA) weld metal compositions. Each composition includes information on 16 elements, including iron (Fe), and six mechanical properties, such as yield strength and tensile strength [5].

Based on this database, numerous studies have analyzed the influence of individual alloying elements on the mechanical properties of welds [6,7,8,9,10]. Traditional statistical techniques such as constraint-based models, multiple regression analysis, and cluster analysis have been employed to explore these relationships. However, the complex interactions between the variables make it challenging to fully untangle these relationships using traditional methods alone.

Artificial neural networks (ANNs) have emerged as a promising alternative to address these issues [11,12]. ANNs are particularly useful in scenarios involving many inputs, outputs, and nonlinear relationships, as they can efficiently perform regression analysis without requiring prior assumptions about the relationships between variables. Consequently, ANNs are effective in predicting the mechanical properties of welds based on their chemical composition. For instance, ANN-based prediction models have been developed for various materials and welding conditions, demonstrating their applicability. Park et al. [13] developed an ANN-based model to predict the yield strength of austenitic stainless steel welds. Sampath [10] proposed an ANN model to predict the Charpy V-notch impact toughness of high-strength steel weld metals based on Evans’s database. Bera and Das [14] developed an ANN model to predict the ultimate tensile strength (UTS), elongation, and Rockwell hardness on the B scale (HRB) for gas metal arc welding (GMAW) of dissimilar steels using current, voltage, and gas pressure as inputs. Payares-Asprino [15] presented an ANN model to predict the yield strength, tensile strength, elongation, and fracture location of duplex stainless steel (SAF 2205) welds in a robotic GMAW process under varying welding conditions. Jung et al. [16] developed an artificial intelligence (AI)-based model to predict the tensile properties of high-strength steels using microstructural factors and chemical compositions. Mezher et al. (2024a) applied various ANN architectures to predict the quality of resistance spot welding (RSW) for AISI 304 stainless steel and quantitatively analyzed the changes in shear strength and nugget diameter according to process parameters [17]. In a subsequent study, Mezher et al. (2024b) investigated dissimilar metal RSW between titanium alloy and AISI 304 austenitic stainless steel using not only ANNs but also Random Forest and CatBoost algorithms and quantitatively assessed the relative importance of input variables [18].

Despite such advantages, ANN models face challenges such as vanishing gradients and overfitting. To overcome these issues, researchers have explored new activation functions and optimization techniques. In addition, recent advancements in deeper networks and effective optimization methods have significantly enhanced model performance. These improvements have enabled the development of robust models that can avoid overfitting and achieve optimal prediction accuracy [19,20,21,22,23,24,25,26,27,28].

First, this study developed a model to predict the mechanical properties of welds based on their chemical compositions, utilizing the experimental database of SMA weld metals published by Dr. Glyn M. Evans [5] and implementing a multilayer ANN structure. Bayesian optimization techniques were used to determine the optimal number of layers and nodes and establish when to stop training. The objective was to create a model that can efficiently predict six mechanical properties of welds using only their chemical compositions.

Next, this research introduced an inverse neural network approach. While traditional methods focus on predicting mechanical properties based on chemical compositions, inverse neural networks can identify combinations of chemical components that achieve specific mechanical properties. This capability is particularly valuable to practitioners and developers who seek to derive the optimal chemical composition to achieve desired mechanical characteristics. To facilitate this, this paper utilized a genetic algorithm (GA) to explore the optimal chemical composition. GAs are powerful tools for discovering optimal combinations by mimicking the principles of natural selection. They also excel at exploring global optima through repeated selection, crossover, and mutation by mimicking the theory of natural selection and evolution. These properties make them well-suited for optimizing complex objective functions defined in high-dimensional spaces, such as those encountered in the chemical composition design problem addressed in this study.

In recent years, hybrid optimization approaches that combine machine learning (ML) techniques with GAs have garnered a significant amount of attention. These combined methods effectively address the limitations of single algorithms and enhance both predictive and exploratory performance. ML-based predictive models can quickly estimate performance within a design space, while GAs help identify combinations that meet specific target conditions through search-based optimization. The integration of these two techniques has been actively applied across various fields, particularly in materials science, where their effectiveness has been demonstrated in alloy composition design. Lee et al. [29] used an ML-aided GA approach to efficiently explore the optimal composition and processing conditions for medium-Mn steel with improved tensile strength and elongation. By integrating data-driven prediction models with GA-based inverse design techniques, ultra-high-strength compositions were successfully obtained. In addition, the effects of trace alloying elements such as Ti, V, and Mo on mechanical properties were investigated through microstructural analysis, demonstrating how an ML–GA-based framework can advance alloy design. Bhat et al. [30] combined GAs with class-based models to simultaneously optimize the strength and elongation of aluminum alloys. The researchers classified different aluminum alloy classes, trained individual regression models for each class, and linked these models with GAs to explore optimal compositions and processing conditions specific to each. Schaufelberger et al. [31] introduced an uncertainty-controlled GA based on ensemble MA predictions to explore the high-dimensional chemical space for designing singlet fission materials. Improving the synergy between ML techniques and GAs is becoming an essential approach to solving inverse design problems [29,30,31,32,33,34,35,36,37,38,39,40,41].

This study proposes a hybrid GA–ANN-based inverse design prediction model that overcomes the limitations of conventional forward-only ANN models by enabling goal-oriented exploration of chemical compositions based on target mechanical properties. The model integrates a high-accuracy ANN, trained on a large-scale weld metal dataset, with a genetic algorithm to iteratively search for optimal compositions that minimize the error between predicted and desired properties. By capturing the complex nonlinear relationships between composition and properties, and automating the optimization process, the framework reduces the need for extensive physical testing, lowers development costs, and improves design efficiency. Repeated validations confirmed its stable and reliable performance, demonstrating potential for broad applicability across various welding processes and alloy design tasks.

This paper is structured as follows: First, it discusses the relevant database and the basic statistical properties of the data. Second, the development of an ANN model for predicting mechanical properties is detailed. Finally, it presents the development of a model utilizing an inverse neural network approach and discusses the results.

## 2. Database

### 2.1. Specifications of the Database

The relationship between chemical composition and mechanical properties of welds was investigated based on a large-scale database of SMA weld metals published by Dr. Glyn M. Evans [5]. Table 1 presents the descriptive statistics of the data, which consists of the chemical composition of 16 elements and their corresponding six mechanical properties. The mechanical properties include yield strength (YS), ultimate tensile strength (UTS), elongation (El), reduction of area (RA), and transition temperature at 100 J and 28 J in the Charpy V-notch test (Temp-100J, Temp-28J). The presented statistical indicators provide fundamental insights into the distribution trends and variability among the characteristics. In particular, descriptive statistics such as the minimum, maximum, mean, and standard deviation of each variable enable a clear understanding of the range and scale of both chemical compositions and mechanical properties. This information is valuable for establishing effective data preprocessing and analysis strategies for the input variables.

### 2.2. Correlation Analysis

**Figure 1:**
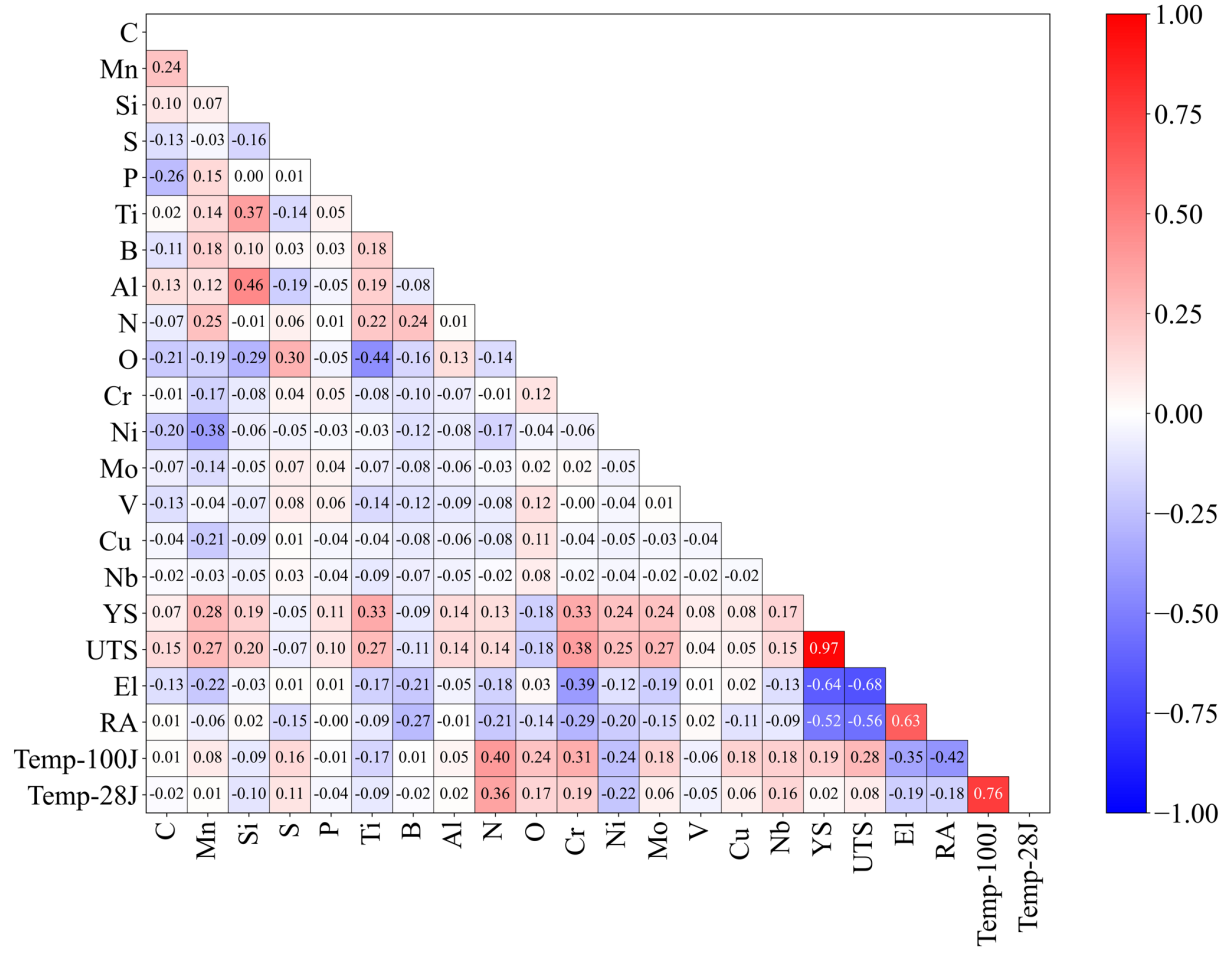
Pearson correlation matrix of chemical composition and mechanical properties.

To assess the approximate correlation between each dataset, Pearson correlation analysis was performed using Equation (1) below, where r is the Pearson correlation coefficient, and x and y are the target variables being analyzed. Figure 1 visually represents the Pearson correlation coefficients between the variables. A value close to 1 indicates a strong positive correlation, while a value close to −1 indicates a strong negative correlation.

(1) $$r=\frac{\sum \left(x_{i}-\bar{x})(y_{i}-\bar{y}\right)}{\sqrt{\sum {\left(x_{i}-\bar{x}\right)}^{2}\sum {\left(y_{i}-\bar{y}\right)}^{2}}}$$

The analysis found that none of the chemical compositions had a correlation coefficient greater than 0.5 with any of the mechanical properties. This outcome is due to the limitations of Pearson correlation analysis, which only examines linear relationships between two variables and is not effective in analyzing nonlinear interactions among chemical composition ratios or between mechanical properties. Specifically, Pearson correlation analysis fails to account for multivariate nonlinear interactions among elemental compositions, multivariate nonlinear interactions between mechanical properties, and multivariate nonlinear interactions between composition ratios and physical properties. To overcome these limitations, this study employs an ANN to analyze multivariate nonlinear interactions.

## 3. Methodology

**Figure 2:**
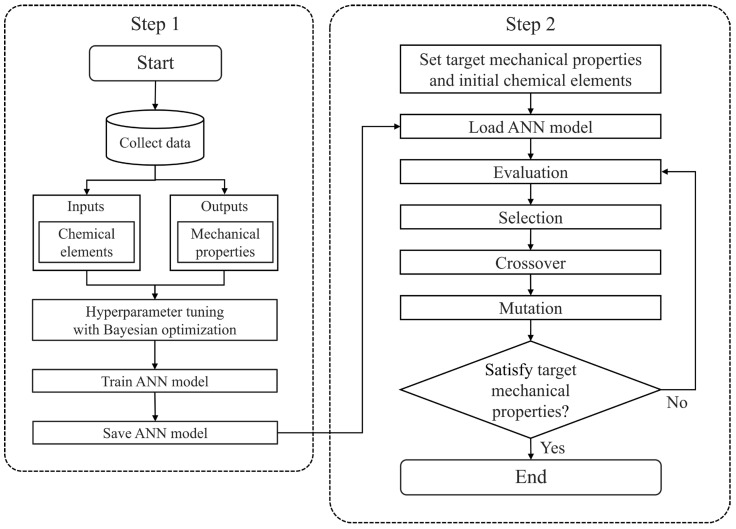
Workflow of the hybrid GA–ANN process.

The hybrid genetic algorithm–artificial neural network (hybrid GA–ANN) applied in this study consists of an ANN prediction model and a GA evaluation model, which combines the powerful predictive capabilities of ANNs with the optimization ability of GAs to facilitate prediction and inverse operations. The overall process involves two main steps, as shown in Figure 2. Step 1 consists of training the ANN, which is used to build a data-driven predictive model. Step 2 involves the process of performing inverse operations based on the trained ANN model to derive the optimal elemental composition ratio that satisfies the target mechanical properties.

In Step 1, the ANN is trained using SMA weld metal data. The elemental composition ratio is set as the input, while the corresponding mechanical properties serve as the output. The model’s hyperparameters, including the number of nodes per hidden layer, the number of hidden layers, and the number of epochs, which are important factors that determine the structure and performance of the neural network, are optimized using Bayesian optimization. The ANN is then trained based on these optimized hyperparameters. The performance of the trained model is evaluated using statistical metrics such as mean squared error (MSE) and the coefficient of determination (R^2^) and finally stored.

In Step 2, after setting and inputting the initial values of the target mechanical properties and elemental compositions, the ANN trained in Step 1 is combined with a GA to identify the combination of elemental compositions that satisfy the target mechanical properties. First, the GA evaluation model predicts the mechanical properties based on the input values of the elemental compositions using the trained ANN. The predicted mechanical properties are compared to the target values, resulting in the elimination of unsuitable elemental compositions while suitable ones are passed on to the next generation. GAs are search algorithms designed to find data adaptable to their environment. Compositions that do not meet the evaluation criteria are discarded, while those that do are optimized through successive generations, retaining their genetic information. During this process, mutation operations are applied to generate new combinations of composition ratios. During mutation, small random changes are introduced to the existing compositions, helping to expand the search space and avoid local optima. Eventually, this process leads to a composition that satisfies the desired mechanical properties.

**Figure 3:**
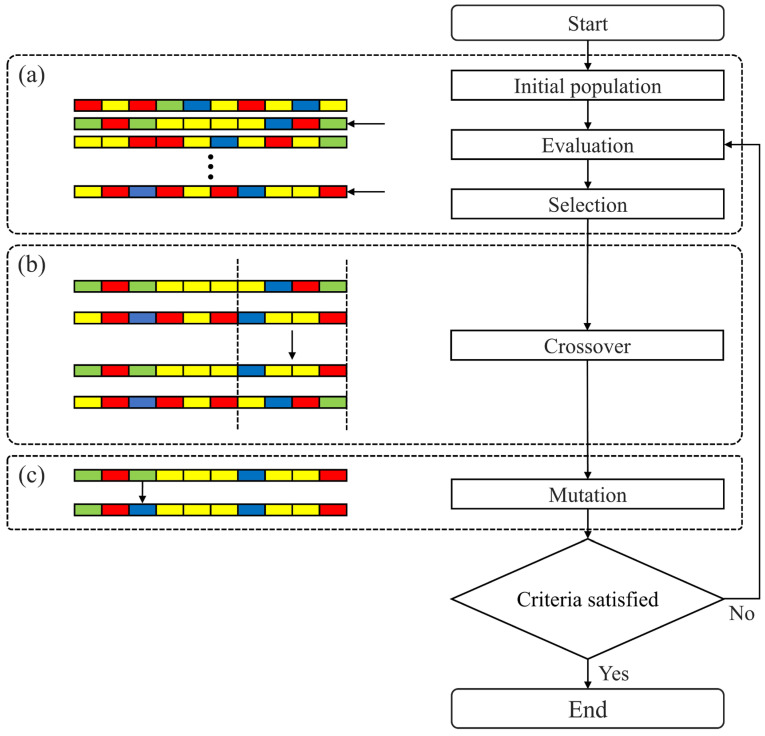
Workflow of the genetic algorithm process.

Figure 3 illustrates the overall workflow of the GA. On the left, a representation of a chromosome shows how chromosomes are structured within the population. On the right, a flowchart describes the main steps of the GA: initialization, evaluation, selection, crossover, and mutation.

Figure 3a depicts the initial stage of the GA. The initial population represents potential solutions in the form of chromosomes. During the evaluation phase, a fitness evaluation determines how suitable each chromosome is for solving the problem. The selection phase then selects the chromosomes that will be passed on to the next generation, where individuals with higher fitness are more likely to be selected.

Figure 3b illustrates how the crossover operation is performed. Selected parent chromosomes exchange genetic material to create new offspring, which increases population diversity and helps expand the search space.

Figure 3c shows the mutation process. In this stage, specific gene materials of certain chromosomes are randomly altered to avoid local optima and facilitate more effective exploration of the search space. Mutation enhances the chances of discovering new optimal solutions, making the overall search process more flexible.

Finally, the GA iterates through the following steps: evaluation, selection, crossover, and mutation. This process continues until the predefined termination criteria are met, which may include reaching an optimal fitness value or exceeding a maximum number of generations. If the termination condition is not satisfied, the process repeats until a satisfactory solution is found.

## 4. Machine Learning Model

### 4.1. Hyperparameter Optimization

The Bayesian optimization method was applied to identify the optimal hyperparameters. This technique utilizes a probability model based on Bayes’s theorem to efficiently explore hyperparameters [19,20]. Bayesian optimization seeks the $x^{+}$ that maximizes the objective function f and is defined as shown in Equation (2), where A is the search range.

(2) $$x^{+}=arg\;\underset{x\in A}{\max}f(x)$$

The core idea of Bayesian optimization is expressed in Equation (3). It shows the relationship between the prior probability $P\left(M\right)$ of an existing model M and the probability $P\left(E|M\right)$ that E will be observed in the model when evidence data E is observed. This process involves updating the posterior probability $P\left(M|E\right)$ during optimization.

(3) $$P\left(M|E\right)\propto \;P\left(E|M\right)P(M)$$

In executing Bayesian optimization, a stochastic model based on a Gaussian process approach is utilized to approximate f and determine the optimal sampling point. This approach balances exploration (testing unknown regions) and exploitation (exploring regions based on the optimal values found in the existing sampling process). The neural network was trained using the Adam (Adaptive Moment Estimation) optimizer, which adaptively adjusts the learning rate for each parameter. The operating principle of this optimization method is described in detail in Section 4.2. The final hyperparameters determined through this process are summarized in Table 2.

### 4.2. Model Structure

This section describes the structure of the ANN. An ANN consists of an input layer, one or more hidden layers, and an output layer, based on a multilayer perceptron structure.

Table 1 shows the data provided to the input and output layers of the ANN. The input data comprises the 16 elemental composition ratios of SMA weld metals, including elements such as carbon (C), manganese (Mn), and silicon (Si). These elements serve as the independent variables the ANN is trained to use for predictions.

The output layer receives the data that the ANN aims to predict, which includes six mechanical properties of the SMA weld metals, as detailed in Table 1. These properties include yield strength (YS), ultimate tensile strength (UTS), elongation (El), reduction of area (RA), and transition temperature at 100 J and 28 J in the Charpy V-notch test (Temp-100J, Temp-28J). These are the dependent variables that the ANN needs to predict.

**Figure 4:**
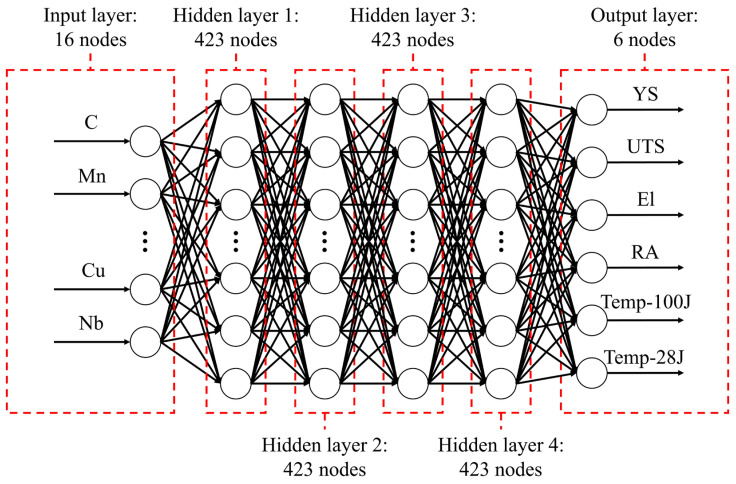
Structure of the prediction model.

Figure 4 illustrates the overall structure of the ANN. To optimize model performance, Bayesian optimization was applied to explore the hyperparameters. The resulting optimal model features four hidden layers, each containing 423 nodes.

In the neural network, the Rectified Linear Unit (ReLU) was used as the activation function. The ReLU function outputs 0 when the input is less than or equal to 0 and returns the input value itself when the input is greater than 0. It is defined as follows in Equation (4):

(4) $$ReLU=max\left(0,x\right)$$

Compared to traditional activation functions such as sigmoid and tanh, ReLU has a simpler computational structure and is effective in alleviating the vanishing gradient problem during training, making it advantageous for stable learning in deep neural networks. For these reasons, ReLU was applied not only to the hidden layers but also to the output layer in this study.

The model was trained using the Adam (Adaptive Moment Estimation) optimizer. Adam is based on stochastic gradient descent (SGD) and estimates both the first moment (mean of the gradients) and the second moment (mean of the squared gradients) for each parameter during training. By automatically adjusting the learning rate for each parameter, Adam suppresses excessive oscillations and facilitates fast and stable convergence.

During the training process, mean squared error (MSE) was used as the loss function. MSE is defined as in Equation (5), where n represents the number of data points, $y_{i}$ is the actual value to be predicted, and ${\hat{y}}_{i}$ is the value predicted by the ANN. This loss function evaluates errors by measuring the mean squared difference between the predicted and actual values.

**Figure 5:**
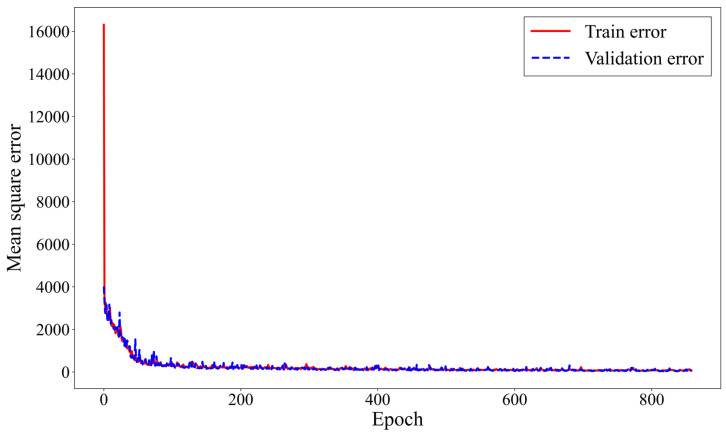
Prediction model performance during training.

Figure 5 shows the MSE progression throughout the training process. The horizontal axis represents the training epochs, and the vertical axis represents the MSE values, with the solid red line indicating the training error and the dashed blue line representing the validation error. As shown in Figure 5, the loss value decreases sharply during the initial training phase and then gradually converges, indicating that the model is moving toward an optimal state and that the training process is stable. Additionally, the training and validation errors remain at similar levels, suggesting that overfitting has not occurred.

(5) $$MSE=\frac{1}{n}\sum_{i=1}^{n}{\left(y_{i}-{\hat{y}}_{i}\right)}^{2}$$

### 4.3. Performance of Machine Learning Model

**Figure 6:**
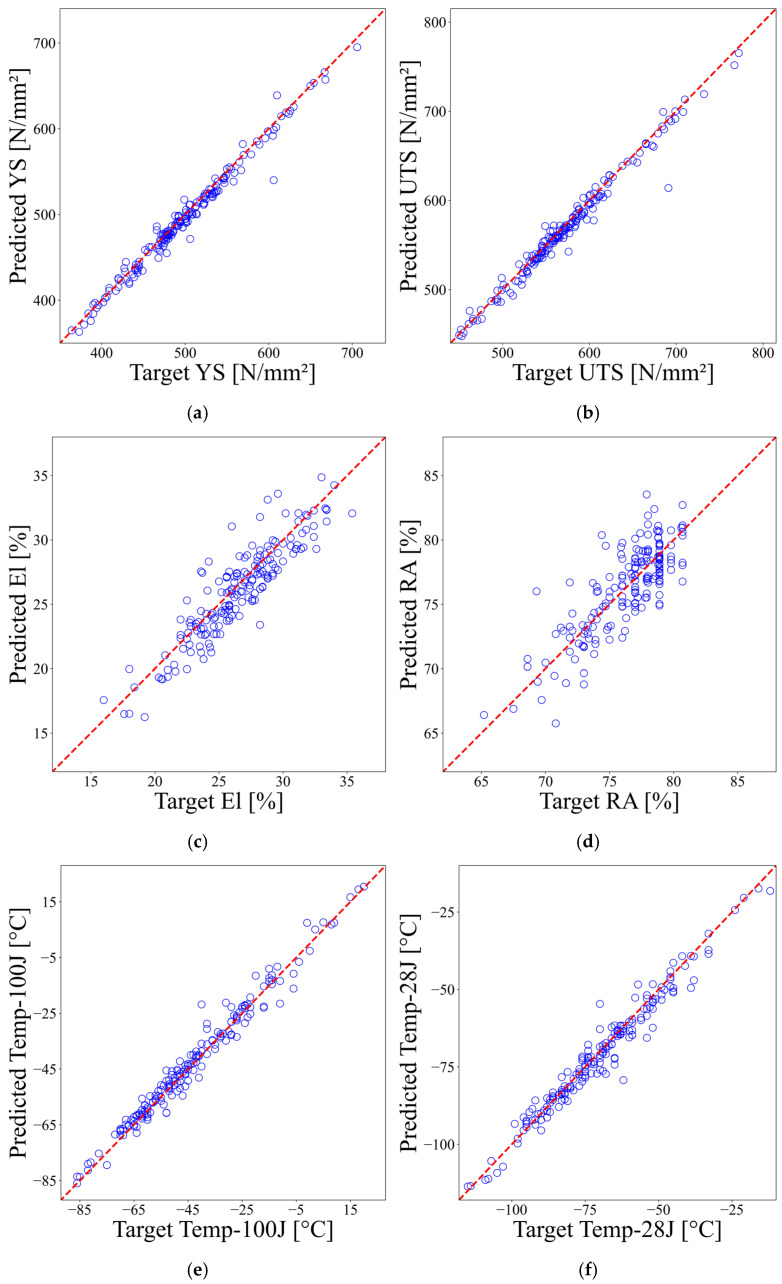
Performance of the prediction model: (**a**) yield strength; (**b**) ultimate tensile strength; (**c**) elongation; (**d**) reduction of area; (**e**) transition temperature at 100 J; (**f**) transition temperature at 28 J.

The performance of the prediction model was evaluated using data that was not included in the training set. Figure 6 displays the performance of the ANN prediction model. The results indicate very high prediction accuracy for yield strength and ultimate tensile strength. The predictions for Temp-100J and Temp-28J show relatively lower accuracy compared to YS and UTS yet still maintain a high level of precision. In contrast, El and RA exhibit large scatters in the predicted data and relatively high errors.

The accuracy of the prediction model was assessed using the coefficient of determination ($R^{2}$) defined in Equation (6). The coefficient of determination reflects how well the regression model explains the variance between the predicted and actual data, with values ranging from 0 to 1. A value closer to 0 indicates weak explanatory power, while a value closer to 1 indicates that the model accurately captures the actual data. The results of the numerical evaluation of the prediction model’s performance using the coefficient of determination are presented in Table 3. The findings indicate that the ANN model, developed from experimental data, is suitable for actual predictions of YS, UTS, Temp-100J, and Temp-28J. However, predicting El and RA proved challenging due to their weak correlation with the chemical compositions.

(6) $$R^{2}=1-\frac{\sum_{i}{\left(y_{i}-{\hat{y}}_{i}\right)}^{2}}{\sum_{i}{\left(y_{i}-\bar{y}\right)}^{2}}$$

### 4.4. Explainable AI with Shapley Values

To quantitatively interpret how the ANN model predicts mechanical properties and to evaluate the relative importance of each input variable (chemical composition), an explainable artificial intelligence (XAI) method based on Shapley values was applied. This approach, grounded in cooperative game theory, fairly assigns each input variable’s contribution to the predicted output, and is known to be effective in enhancing model interpretability even in complex multivariate regression problems [42,43].

Shapley values mathematically define the importance of each variable by calculating its average marginal contribution to the model’s output. This can be expressed as follows:

(7) $$\varphi_{i}\left(f,x\right)=\sum_{S}\frac{\left|S\right|!\left(\left|N\right|-\left|N\right|-1\right)!}{\left|N\right|!}\left[f\left(S⋃\left\{i\right\}\right)-f\left(S\right)\right]$$

Here, $\varphi_{i}$ denotes the Shapley value of feature i, N represents the set of all features, S is any subset of features excluding i, and $f\left(S\right)$ refers to the model prediction based solely on the input subset S. This formula calculates the average contribution of feature i to the model prediction across all possible combinations in which it is newly added. It provides a fair and consistent explanation even in the presence of interactions among variables.

**Figure 7:**
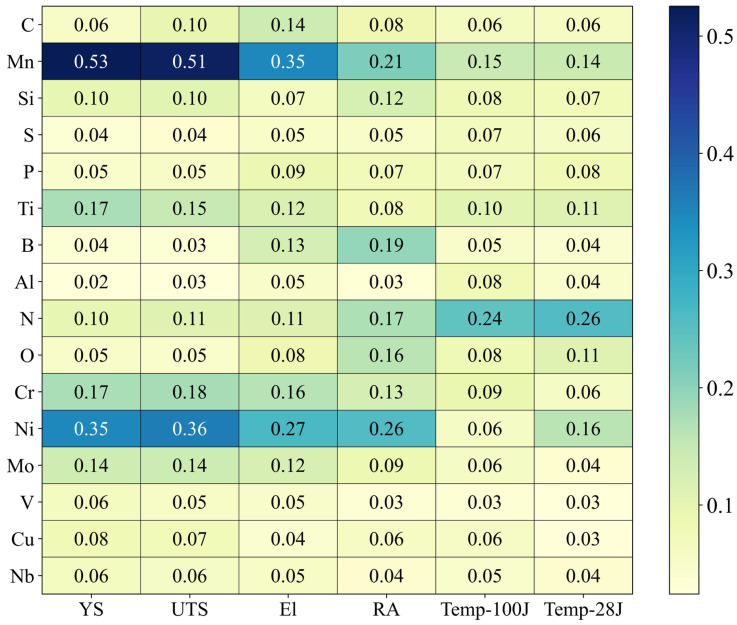
Shapley values representing the contribution of each chemical element to the prediction of mechanical properties.

Figure 7 presents a heatmap visualizing the average absolute Shapley values of 16 chemical elements across six predicted mechanical properties, indicating the extent to which each element contributes to the predictions.

The analysis revealed that Mn exhibited the highest contribution in predicting YS and UTS, confirming its critical role in determining the strength characteristics of weld metals. Ni also showed consistently high importance across all mechanical properties, with particularly notable contributions to the predictions of El, RA, and Temp-100J.

In contrast, N demonstrated high Shapley values for properties related to transition temperature, such as Temp-100J and Temp-28J, which are defined based on specific absorbed energies in Charpy V-notch impact tests. This suggests that N is an important factor in enhancing toughness. On the other hand, elements such as S, P, and Al generally exhibited low contributions, indicating that their influence on the model’s predictions is relatively minor.

These results demonstrate that the ANN model does not function merely as a black box predictor but rather learns patterns consistent with metallurgical mechanisms. Furthermore, the findings provide valuable quantitative evidence for prioritizing alloying elements during the inverse design process, thereby improving design efficiency.

## 5. Result of Inverse Prediction Model

Once the ANN model describing the relationship between the chemical composition and mechanical properties of the weld is complete, it can be combined with a GA to explore combinations of chemical compositions that meet the target mechanical properties, as illustrated in Step 2 of Figure 2.

To apply the hybrid GA–ANN-based inverse prediction model, the target mechanical properties must first be determined. The case settings for the target mechanical properties used in this study are summarized in Table 4.

The key parameters of the GA were set as follows. The population size was set to 100 to allow for a diverse range of composition combinations to be explored in each generation. The crossover probability was set to 0.8 to promote active recombination between parent chromosomes, while the mutation probability was set to 0.1 to prevent premature convergence to local optima and to ensure diversity in the search space. The algorithm was configured to run for a maximum of 100 generations, during which optimal composition combinations were iteratively derived. Table 5 summarizes the detailed GA parameters used in this study.

After determining the mechanical properties, the next step is to load the pre-trained ANN model. This model follows the structure shown in Figure 4, where the chemical composition of the weld is entered into the input layer, and the corresponding mechanical properties are obtained from the output layer. The loaded ANN model acts as a black-box objective function within the inverse prediction model. It is important to note that since the trained ANN model acts as an objective function, it explores chemical composition combinations under the assumption of 100% prediction accuracy.

Each chromosome used in the GA consists of 16 types of weld chemical compositions. When a chromosome is input into the ANN, the mechanical properties for that particular combination are output. The relationship between the output properties and the pre-set target mechanical properties is evaluated using an L1 loss-based fitness function, as shown in Equation (8), where ${\hat{y}}_{i}$ represents the mechanical properties predicted by the inverse prediction model, and $y_{i}\;$represents the pre-set target mechanical properties.

(8) $$L1\;loss=\sum_{i=1}^{n}\left|y_{i}-{\hat{y}}_{i}\right|$$

**Figure 8:**
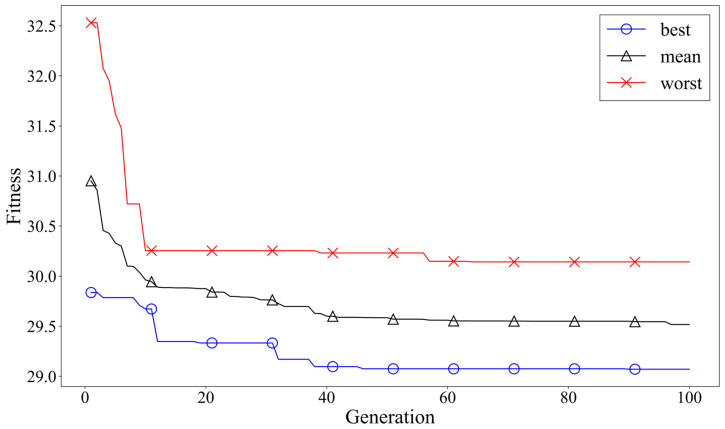
Fitness performance of the hybrid GA–ANN inverse prediction model over generations.

Figure 8 shows the evolution of the fitness value over the generations of the GA. The best and worst fitness values from 10 iterations of the inverse prediction are presented, along with the average value of these iterations, providing an overview of the trends observed during repeated trials.

This analysis enables the identification of the chromosome with the smallest error between the predicted output and the target material properties, which is considered the optimized chemical composition to achieve the desired mechanical properties. The results of these iterations are summarized in Table 6.

**Figure 9:**
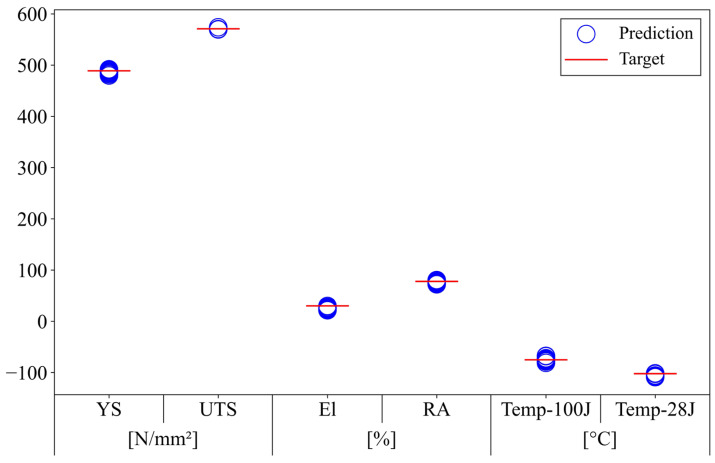
Mechanical properties corresponding to the suggested compositions.

Figure 9 visualizes the distribution of predicted mechanical properties by inputting the chemical compositions listed in Table 6 into the pre-trained ANN model. For each property, the target values are labeled, and the predicted property values over 10 trials are circled. The performance of the hybrid GA–ANN-based inverse prediction model was validated by assessing how well each chemical composition satisfied the target properties.

## 6. Discussion

The hybrid GA–ANN framework proposed in this study has shown promise in effectively solving inverse design problems in welding alloy design. While traditional statistical analyses or individual ML models have proven effective in predicting properties for given compositions, they are limited in solving the inverse problem of deriving an optimal composition based on targeted material properties. This study overcomes these limitations by combining the predictive capabilities of ANNs with the optimization capabilities of GAs.

The ANN prediction model exhibited high accuracy, especially in key strength properties such as YS and UTS, while also maintaining a high level of prediction accuracy for Temp-100J and Temp-28J. This indicates that the influence of the chemical composition of SMAW welds on these properties can be effectively captured. Conversely, the prediction accuracy for El and RA was somewhat lower, as these properties are influenced by various factors, including welding process variables, cooling rates, and microstructure formation, and cannot be explained by chemical composition alone. This suggests the need to include additional physical variables or microstructure data for future model improvements.

To interpret the prediction results and ensure model transparency, this study applied an XAI approach based on Shapley values. This method enabled the quantitative evaluation of how much the ANN model relies on each chemical element when predicting mechanical properties. The analysis showed that Mn and Ni made the largest contributions to the predictions of YS and UTS, while N played a significant role in the predictions of Temp-100J and Temp-28J. These findings demonstrate that the ANN model can be extended beyond a traditional black-box predictor to an interpretable model through Shapley value-based analysis, where the contribution of each input variable can be quantitatively assessed. This enhances the model’s reliability and transparency, reinforcing its potential as a decision-support tool in practical alloy composition design.

The GA-based inverse design process gradually converged to the optimal solution through repeated generation changes and mutation operations, with consistent results observed over 10 iterations. During this process, the L1 loss-based fitness function effectively minimized the absolute error between the target and predicted values, while the mutation operation helped prevent the algorithm from falling into a local minimum. Notably, several trials yielded compositional combinations that closely matched the target properties, demonstrating the reliability and practicality of the proposed framework.

The GA–ANN-based approach proposed in this study can serve as an effective tool for exploring global optima in composition design problems. However, alternative optimization algorithms such as Particle Swarm Optimization (PSO) and Bayesian inverse design methods may offer relative advantages in terms of convergence speed and quantification of predictive uncertainty. In future work, we plan to develop this framework further by comparing the performance of various optimization techniques depending on the problem characteristics, with the aim of identifying the most suitable algorithmic combinations. In particular, comparative studies with advanced evolutionary algorithms such as L-SHADE and EBLSHADE are also being considered as future research directions.

Furthermore, this study aimed not only to evaluate model performance but also to consider its potential application in real-world manufacturing environments. Defining target properties and identifying compositions that meet these requirements are closely related to the development of welding materials and prototype design. The approach of this study effectively reflects these practical needs. Such a model can serve as an intuitive and valuable design tool for both researchers and industry and is expected to significantly contribute to reducing the design cost and time for high-performance materials.

## 7. Conclusions

This study developed a hybrid AI-based model that can simultaneously predict the mechanical properties and perform inverse design of SMAW weld metals. First, based on a large-scale database provided by Dr. Glyn M. Evans, a multilayer ANN model was constructed, using the chemical composition of 16 elements as input and six mechanical properties as output. Bayesian optimization was used to fine-tune the model structure, resulting in good prediction accuracy.

Additionally, this study demonstrated the interpretability of the ANN model’s predictions through an explainable AI analysis based on Shapley values. By quantitatively assessing the importance of each input element, the proposed approach contributes to enhancing the model’s reliability and its practical applicability as a design tool.

Next, an inverse design framework was constructed by combining a GA with the trained ANN model. The GA iteratively refined the elemental composition to minimize the error between the target mechanical properties and the ANN predictions, thereby exploring various optimal combinations. In all 10 iterations of the inverse prediction, the compositional combinations closely matched the target properties, demonstrating the model’s stability and effectiveness.

This hybrid GA–ANN approach represents a significant turning point beyond traditional composition-property modeling, enabling goal-oriented weldment design. Particularly in the early stages of weldment development, this model serves as a powerful tool to reduce experiment-based trial and error while also improving design efficiency and cost-effectiveness. Furthermore, the methodology presented in this study can be applied not only to SMAW but also to various welding methods, such as GMAW and GTAW, and dissimilar materials. It is expected to be widely applicable in various fields, including the development of new high-performance alloys and the design of materials for extreme environments.

Several considerations should be taken into account for future studies. First, in addition to chemical composition, process variables such as heat treatment conditions, cooling rate, and welding current should be included as inputs to increase the model’s explanatory power. Second, efforts should be made to improve the accuracy of physical property predictions by integrating microstructure data and ensuring the physical validity of result interpretations. Finally, after deriving the optimal composition, it is necessary to conduct stepwise studies to examine the model’s applicability in practice through specimen fabrication and experimental validation.

## Figures and Tables

**Table 1:** Summary of chemical composition and mechanical properties.

Element	Min. Value	Max. Value	Mean Value	Std. Dev.	Unit
C	0.035	0.152	0.071	0.011	wt%
Mn	0.23	2.1	1.262	0.393	wt%
Si	0.001	1.11	0.352	0.104	wt%
S	0.003	0.046	0.007	0.002	wt%
P	0.003	0.04	0.008	0.003	wt%
Ti	1	770	115.541	152.679	ppm
B	1	200	21.387	44.732	ppm
Al	1	680	40.307	111.877	ppm
N	35	270	92.916	47.832	ppm
O	217	1535	397.230	87.849	ppm
Cr	0.026	3.5	0.138	0.447	wt%
Ni	0.03	5.48	0.295	0.982	wt%
Mo	0.005	1.16	0.050	0.201	wt%
V	3	2873	48.924	237.194	ppm
Cu	0.02	2.04	0.071	0.235	wt%
Nb	3	980	19.046	84.891	ppm
YS	310	1026	505.589	77.196	N/mm^2^
UTS	345	1123	578.349	77.657	N/mm^2^
El	7.4	35.8	26.009	3.783	%
RA	10.8	87.8	75.719	4.608	%
Temp-100J	−89	45	−43.265	23.500	°C
Temp-28J	−145	77	−69.162	23.956	°C

**Table 2:** Optimized hyperparameters.

Hyperparameters	Values
Number of nodes per hidden layer	423
Number of hidden layers	4
Epochs	859

**Table 3:** Performance evaluation of the prediction model.

Element	YS	UTS	El	RA	Temp-100J	Temp-28J
	0.9805	0.9778	0.8208	0.6685	0.9739	0.9620

**Table 4:** Case settings for target mechanical properties.

Element	YS (N/mm^2^)	UTS (N/mm^2^)	El (%)	RA (%)	Temp-100J (°C)	Temp-28J (°C)
	489	571	30.2	78	−75	−102

**Table 5:** Genetic algorithm parameters.

Parameters	Values
Population size	100
Crossover probability	0.8
Mutation probability	0.1
Maximum number of generations	100

**Table 6:** Optimized chemical compositions generated by the genetic algorithm.

Element	Trial 1	Trial 2	Trial 3	Trial 4	Trial 5	Trial 6	Trial 7	Trial 8	Trial 9	Trial 10	Unit
C	0.035	0.038	0.038	0.035	0.035	0.041	0.038	0.037	0.039	0.035	wt%
Mn	0.914	0.846	1.334	1.887	1.142	0.277	0.242	1.592	0.589	1.202	wt%
Si	0.731	1.081	0.823	0.437	0.926	0.036	0.016	0.648	0.019	0.033	wt%
S	0.0051	0.003	0.003	0.003	0.0073	0.003	0.003	0.008	0.003	0.0034	wt%
P	0.003	0.006	0.003	0.008	0.003	0.003	0.003	0.003	0.009	0.003	wt%
Ti	301.44	79.08	497.91	27.84	402.34	357.53	283.78	533.76	48.53	670.27	ppm
B	175.5	9.03	121.14	4.71	185.06	167.14	70.11	143.32	34.99	130.49	ppm
Al	256.44	56.83	558.31	5.13	262.96	35.41	25.38	411.21	5.27	155.95	ppm
N	120.38	111.47	136.52	152.45	152.41	195.66	44.95	227.48	126.85	249.63	ppm
O	283.86	235.56	253.61	273.91	254.92	304	260.96	220.8	423.59	244.35	ppm
Cr	0.061	0.065	0.245	0.262	0.091	0.104	0.062	0.036	0.104	0.266	wt%
Ni	2.732	0.078	4.020	0.254	0.048	0.058	0.058	2.743	1.933	0.061	wt%
Mo	0.055	0.121	0.062	0.522	0.041	0.024	0.030	0.017	0.040	0.005	wt%
V	119.15	25.26	24.23	530.65	191.64	307.96	144.5	27.04	356	122.74	ppm
Cu	0.13	0.02	0.7	0.12	0.22	0.08	0.02	0.34	0.06	0.08	wt%
Nb	7.32	539.03	12.17	7.15	19.69	3.05	630.31	62.81	5.72	43.33	ppm

## Data Availability

The data presented in this study are available on request from the corresponding author.
